# High end of life health care costs and hospitalization burden in inflammatory bowel disease patients: A population-based study

**DOI:** 10.1371/journal.pone.0177211

**Published:** 2017-05-12

**Authors:** Sanjay K. Murthy, Paul D. James, Lilia Antonova, Mathieu Chalifoux, Peter Tanuseputro

**Affiliations:** 1 Department of Medicine, Division of Gastroenterology, The Ottawa Hospital and University of Ottawa, Ottawa, Ontario, Canada; 2 Ottawa Hospital Research Institute, Ottawa, Ontario, Canada; 3 Institute for Clinical Evaluative Sciences (ICES uOttawa), Ottawa, Ontario, Canada; 4 Bruyère Research Institute, Ottawa, Ontario, Canada; National Cancer Institute, UNITED STATES

## Abstract

**Background:**

End of life (EOL) care is associated with greater costs, particularly for acute care services. In patients with inflammatory bowel disease (IBD), EOL costs may be accentuated due to reliance on hospital-based services and expensive diagnostic tests and treatments. We aimed to compare EOL health care use and costs between IBD and non-IBD decedents.

**Methods:**

We conducted a retrospective cohort study of all decedents of Ontario, Canada between 2010 and 2013 using linked health administrative data. IBD (N = 2,214) and non-IBD (N = 262,540) decedents were compared on total direct health care costs in the last year of life and hospitalization time during the last 90 days of life.

**Results:**

During the last 90 days of life, IBD patients spent an average of 16 days in hospital, equal to 2.1 greater adjusted hospital days (95% confidence interval [CI] 1.5–2.8 days) than non-IBD patients. IBD diagnosis was associated with $7,210 CAD (95% CI $5,005 - $9,464) higher adjusted per-patient cost in the last year of life, of which 76% was due to excess hospitalization costs. EOL cost of IBD care was higher than 15 of 16 studied chronic conditions. Health care costs rose sharply in the last 90 days of life, primarily due to escalating hospitalization costs.

**Conclusions:**

IBD patients spend more time in hospital and incur substantially greater health care costs than other decedents as they approach the EOL. These excess costs could be curtailed through avoidance of unnecessary hospitalizations and expensive treatments in the setting of irreversible deterioration.

## Introduction

Crohn's disease and ulcerative colitis are chronic inflammatory bowel diseases (IBD) that afflict 0.67% and 0.5% of the Canadian and U.S. populations, respectively.[1;2] Patients with IBD experience substantial morbidity from their disease and use a considerable amount of health care resources.[2;3] Population-based studies estimate the direct annual health care costs of IBD at CAD 1.2 billion in Canada and $6.3 billion USD in the U.S.[2;4] Hospital admissions and medications account for the greatest burden of direct health care costs in IBD patients.[2;4–6]

The end-of-life (EOL) period for an individual represents a time of rapidly rising health care demands.[7–9] Health care services provided in decedents’ last year of life have being measured to account for approximately 10% ($4.7 billion CAD) of the annual health care budget of Ontario, Canada’s largest province, even though decedents constitute only 0.67% of the Canadian population.[4;10] The EOL period is, thus, an important target for the development of cost-effective strategies for health care delivery.

EOL health care use and costs have not been characterized in IBD patients. Given the high cost of many newer drugs used to treat IBD and the frequent reliance on hospital-based care, it is conceivable that health care costs and acute care use at the EOL may be higher in IBD patients than in other decedents. Therefore, EOL care would constitute an important target for the development of cost-effective care strategies among IBD patients. In this study, we aimed to characterize EOL direct health care costs and health care utilization across sectors among IBD decedents, and to compare this to non-IBD decedents from the general population.

## Materials and methods

### Study design and outcomes

We conducted a retrospective population-based cohort study of all individuals who died in the province of Ontario, Canada, between April 1, 2010 and March 31, 2013. We excluded decedents who did not have at least one record of health care use in Ontario within 365 days of death. Ontario is Canada’s largest province, comprising more than 13 million inhabitants, and consists of a largely single payer health care system, in which all necessary medical services are publicly-funded through the Ontario Ministry of Health and Long-term Care (MOHLTC). Most hospital and ambulatory-based health care services are covered for all residents through this program. Prescription drugs, however, are only covered for individuals 65 years or older and those requiring social assistance, as well as for very expensive drugs that exceed the pre-determined deductible for individuals requiring these treatments. Some out-of-hospital services, such as allied health services (i.e. physiotherapy, occupational therapy, massage therapy and dietetic services), dental procedures, cosmetic and elective procedures and over the counter medications, are not paid for by the public health system.

Identified decedents with IBD were compared to non-IBD decedents on the following measures: (i) total and sector-specific direct health care costs in the last year of life; (ii) health care cost trajectory in the last year of life; (iii) costs and use of specific health services during the last 90 days of life, including costs of hospitalizations associated with intensive care unit (ICU) stay or major abdominal surgery (MAS), number of gastrointestinal (GI) endoscopic studies and number of major body imaging studies (to explore potential explanatory factors for any observed difference in direct health care costs between groups); and (iv) time spent in high-level institutional care during the last 90 days of life, particularly acute hospital care. We additionally compared direct health care costs in the last 90 days of life to the first 90 days in the last year of life. Furthermore, to establish a benchmark for comparison, we evaluated mean health care costs in the last year of life among patients with 16 other complex chronic diseases and provide a breakdown of health care sector costs for patients with five conditions that are associated with high morbidity and mortality–cancer, chronic obstructive pulmonary disease (COPD), congestive heart failure (CHF), diabetes mellitus (DM) and renal disease.

### Data sources and study definitions

We linked multiple Ontario health administrative datasets at the individual level to conduct this study. These datasets were linked using unique encoded identifiers and analyzed at the Institute for Clinical Evaluative Sciences (ICES).[11] Specific datasets that were used in this study are presented in S1 Table. All legal long-term residents of Ontario (>99% of the population) receive complete coverage for medically necessary services from the provincial government, which enables comprehensive tracking of health care encounters. All diagnostic and procedural information is coded using the International Classification of Diseases (ICD) and Canadian Classification of Interventions (CCI) nomenclature, respectively.

We identified IBD patients from among Ontario decedents by linking vital statistics data from the Registered Persons Database (RPDB) to the Ontario Crohn’s and Colitis Cohort (OCCC). The OCCC is a database of IBD patients living in Ontario that was created using validated algorithms of IBD-related health care contacts.[12;13] These algorithms demonstrated a sensitivity of 92.3% and specificity of 99.1%.[12;13] All decedents that were not identified in the OCCC were assigned to the non-IBD comparator group.

We further identified decedents with one of 16 complex chronic diseases, using either validated case ascertainment algorithms for six of the 16 chronic conditions: Asthma,[14] COPD,[15] CHF,[16] Dementia,[17] Diabetes[18] and Hypertension.[19] Where a validated algorithm did not exist, using a similar algorithm to what was used to derive these validated cohorts (at least one inpatient or two outpatient diagnoses within a two-year period).[20] The 16 conditions were selected based on their high cost and prevalence in society.

We identified primary and secondary causes of death from the Ontario Registrar General Database (ORGD). We used census data to acquire information on patient income (categorized as quintiles of median neighbourhood household income) and residential setting (rural [less than 10,000 individuals] vs. urban). We assessed co-morbidity burden by a weighted score based on the number of Johns Hopkins aggregated diagnostic groups (ADG),[21;22] using hospital admissions and outpatient physician visits for the two-year pre-study period. Hospitalizations were ascertained from the Canadian Institutes of Health Information Discharge Abstract Database (CIHI-DAD). Outpatient encounters were identified from the Ontario Health Insurance Plan (OHIP) database using unique physician billing claims. Physicians’ claims data was also used to capture MAS potentially relevant to IBD, gastrointestinal endoscopic procedures and advanced radiographic imaging studies (S2 Table).

To derive individual-level direct health care costs at the EOL, we used a costing algorithm developed for use with Ontario health administrative databases (HADs), which assigns unit costs for specific types of health care use across various sectors of health care expenditures.[23] These are then summed to obtain annual estimates of total direct health care costs for each individual (S1 Table). Out-of-pocket costs, costs paid for by private insurers (e.g., co-payment for medications and accommodation, devices, and private home care), costs relating to public health services, certain capital costs and indirect health care costs (i.e. due to lost productivity) are not included in this calculation. Overall, the costing algorithm captures about 80% of total direct public payer health care costs.

### Statistical analyses

Bivariate comparisons between IBD and non-IBD decedents were conducted using the student’s t-test (for normally distributed interval variables), Wilcoxon rank-sum test (for non-normally distributed interval variables) and chi-square test (for categorical variables). Paired t-tests were used for bivariate comparisons of total direct health care costs within subjects for different time periods prior to death. After confirming that outcomes followed a normal distribution, multiple linear regression analysis was used to compare number of days spent in acute care institutions in the last 90 days of life and total direct health care costs in the last year of life between IBD and non-IBD patients. All models included IBD status, age, sex, household income, residential setting and co-morbidity burden as additional covariates and tested for interaction between age and IBD status. Individuals with missing observations were excluded in the multivariable analysis. Statistical significance was defined as a p-value of < 0.05 based on two-tailed hypothesis testing. All analyses were performed using SAS 9.3 (SAS Institute Inc., Cary, NC).

We conducted a sensitivity analysis to assess the potential impact of acute causes of death unrelated to underlying chronic disease on study outcomes, by excluding individuals whose primary cause of death was attributable to an external cause or to a complication of pregnancy or the puerperium, based on relevant ICD-10 diagnostic categories (XV, XVI, XVIII, XIX, XX). In a second sensitivity analysis, we excluded the costs of prescription drugs and devices from total direct costs, as they are only captured for individuals over the age of 65 and those requiring government financial assistance.[24]

### Ethical considerations

This study was approved by the research ethics board at Sunnybrook Health Sciences Centre, Toronto, Canada. The administrative databases employed in the study rely on coded data. Personal identifiers are not provided to researchers. Therefore, patient privacy was not a concern in this study.

## Results

### Study patients

We studied 264,754 decedents (98.9% of all Ontario decedents), of which 2,214 (0.83%) had IBD, including 975 with Crohn’s disease and 1,114 with ulcerative colitis. Characteristics of study patients at the time of death are shown in Table 1. Roughly 80% of decedents were 65 years of age or older and close to 85% lived in an urban setting. More than 96% had at least one chronic condition and greater than 75% had 3 or more chronic conditions at the time of death. IBD was listed as a “most responsible” or “co-morbid” cause of death in 11.6% of IBD decedents.

**Table 1:** Baseline characteristics of study patients.

	Non-IBD N (%)	IBD N (%)	p-value (IBD vs. non-IBD)
**Age**			
Median (IQR)	80 (68–87)	76 (63–85)	<0.0001
**Sex**			
Female	134,491 (51.2)	1,143 (51.6)	0.7083
Male	128,049 (48.8)	1,071 (48.4)	
Income Quintile^**a**^			
Q1	59,591 (22.7)	474 (21.4)	0.0070
Q2	54,380 (20.7)	464 (21.0)	
Q3	50,040 (19.1)	431 (19.5)	
Q4	48,812 (18.6)	400 (18.1)	
Q5	46,017 (17.5)	429 (19.4)	
Missing	3,700 (1.41)	16 (0.72)	
**Residential Setting**			
Urban	221, 492 (84.4)	1,902 (85.9)	< .0001
Rural	37,967 (14.5)	309 (14.0)	
Missing	3081 (1.17)	3 (0.14)	
**# Chronic Conditions**^**b**^			
0	9565 (3.64)	33 (1.49)	<0.0001
1	18,402 (7.01)	140 (6.32)	
2	31116 (11.85)	241 (10.89)	
3	44, 429 (15.78)	312 (14.09)	
4	43,889 (16.72)	412 (18.61)	
5+	118139 (45.00)	1,076 (48.60)	

^a^ Reflects median neighbourhood household income quintile for pre-defined geographic area.

^b^ Includes coronary artery disease (with acute myocardial infarction), stroke, peripheral vascular disease, congestive heart failure, arrhythmia, chronic obstructive pulmonary disease, asthma, renal disease, hypertension, diabetes mellitus, cancer, dementia, depression, osteoporosis, osteoarthritis and other arthritis; excludes IBD.

Baseline characteristics were fairly comparable between IBD and non-IBD decedents. IBD decedents were slightly younger (median age: 76 vs. 80, p = < 0.0001), had slightly higher co-morbidity burden (median number of ADG groups: 12 vs. 11, p = < .0001; median number of chronic diseases: 4.0 vs. 4, p = < .0001) and had marginally greater representation in higher income groups (global p = 0.0167).

### Total and sector-specific direct health care costs in the last year of life

Mean individual direct costs of care and health care usage in the last year of life across health care sectors are shown in Table 2. Hospitalization costs, physicians’ billings and prescription drugs and devices accounted for 44.1%, 9.9% and 5.5% of total direct health care costs, respectively. On average, each IBD patient incurred an additional $12,708 CAD (3.7%) in direct costs during the last year of life as compared to a non-IBD patient ($66,263 vs. $53,555 CAD). Of this, $9,701 (76.3%) was attributable to excess hospitalization costs, of which $7,521 (77.5% of hospitalization costs) was attributable to hospitalizations associated with 1 or more ICU stays. Overall, hospitalization costs were 42.3% greater per IBD decedent than non-IBD decedent, even though only 7.6% more IBD patients required hospitalization.

**Table 2:** Mean health care sector costs per patient and frequency of health care sector usage in the last year of life among IBD and non-IBD decedents.

	Non-IBD Decedents (N = 262,540)		IBD Decedents (N = 2,214)	
Health Care Sector	Mean Cost per decedent ($CAD)	Number of Users (% of decedents)	Mean Cost per decedent ($CAD)	Number of Users (% of decedents)
All-Cause Hospitalization	22,928	195,509 (74.5%)	32,629	1,817 (82.1%)
Hospitalization without ICU Stay	12,142	139,820 (53.3%)	14,322	1,197 (54.1%)
Hospitalization with ≥ 1 ICU Stay	10,786	55,689 (21.2%)	18,307	620 (28.0%)
Emergency Department	1,270	217,587 (82.9%)	1,623	1,955 (88.3%)
Complex Continuing Care	3,436	29,501 (11.2%)	3,702	274 (12.4%)
Rehabilitation	884	9,851 (3.8%)	1,579	130 (5.9%)
Long-term Care	8,344	63,710 (24.3%)	5,720	375 (16.9%)
Home Care	4,421	158,124 (60.2%)	5,356	1,492 (67.4%)
Outpatient clinics	3,477	86,494 (32.9%)	3,813	986 (44.5%)
Physician Billings	5,326	258,126 (98.3%)	7,037	2,206 (99.6%)
Non-physician Billings (OHIP)	323	128,209 (48.8%)	251	1,035 (46.7%)
Laboratory (OHIP)	214	207,598 (79.1%)	266	1,874 (84.6%)
Drugs/Devices	2,931	233,682 (89.0%)	4,287	1,988 (89.8%)
**Total Cost**	53,555	259,500 (98.8%)	66,263	2,212 (99.9%)

All costs are in 2013 Canadian dollars. Per-person health care sector costs were averaged across all decedents (users and non-users) within each group. P-value <0.0001 for all comparisons of health sector costs between IBD and non-IBD patients, except complex continuing care (p = 0.073).

Health care expenditures were also greater for IBD patients in all sectors except for long-term care, in which costs were almost 46% higher for non-IBD patients ($5,720 vs. $8,344 CAD). Individual health care costs were higher for Crohn’s disease than for ulcerative colitis ($70,408 vs. $63,655 CAD), largely due to excess hospitalization costs ($35,983 vs. $30,471 CAD). Ambulatory drug costs among patients over the age of 65 and those receiving social assistance accounted for 6.5% of public health care costs among IBD decedents. Less than 2.3% of IBD decedents received a biologic agent in the last year of life (infliximab, adalimumab, certolizimab-pegol or golimumab), and this treatment accounted for roughly 10.5% of public health care drug costs among IBD decedents.

After adjusting for socio-demographic factors and co-morbidity burden, IBD diagnosis was associated with $7,210 CAD (95% confidence interval [CI] $4,975 - $9,446) greater cost per person during the last year of life (Table 3). Excluding the 5.5% of patients who died from acute external causes or complications of pregnancy or the puerperium, IBD diagnosis was still associated with $7,150 (95% CI $4846 - $9454) greater cost during the last year of life. Exclusion of prescription drugs and devices from overall costs reduced the excess costs associated with IBD slightly to $6,008 CAD (95% CI $3,790 - $8,2225). The observed associations did not differ significantly by age group in any of the analyses (interaction term between age and disease status non-significant in multivariable analysis).

**Table 3:** Multiple linear regression model of health care costs per person in the last year of life.^a^

Variable	Parameter Estimate (95% CI)
IBD (vs. no IBD)	7, 210 (4,975, 9446)
Age Group	
<19	33, 965 (30,9437, 36,988)
19–44	2228 (886.6, 3,570)
45–54	150.2 (-938.3, 1,239)
65–74	-2,046 (-2,853, -1,241)
75–84	-8,146 (-8,885, -7,408)
85–94	-12,595 (-13,332, -11,857)
95+	-10,895 (-11,957, -9,834)
(Ref 55–64)	
Female Sex (vs. Male)	4,517 (4,099, 4,935)
Income Quintil	
Q1	739.1 (108.5, 1,370)
Q2	316.8 (-326.9, 960.6)
Q4	613.7 (-47.17, 1275)
Q5	327.6 (-344.4, 999.6)
(Ref Q3)	
Urban resident (vs. Rural)	-3773 (-4,354, -3,192)
# ADG Groups	1,298 (1,284, 1,312)

^a^ Costs in 2013 Canadian dollars.

### End of life health care costs in IBD compared to other chronic diseases

**Fig 1:**
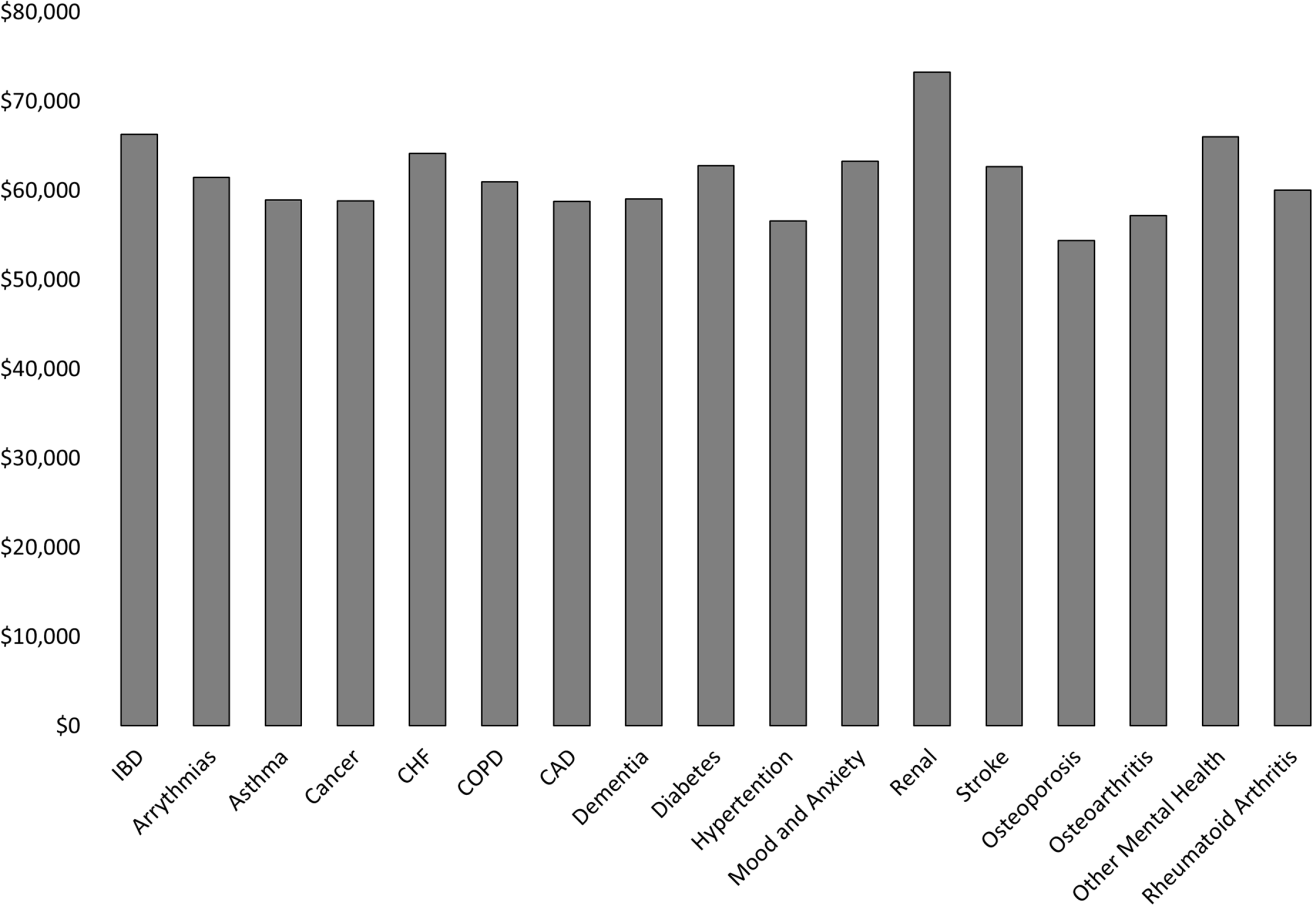
Mean direct health care costs per patient in the last year of life across various chronic diseases.

In the last year of life, IBD patient care incurred 8.4% higher costs than the mean of 16 other complex chronic diseases ($66,263 vs. $61,142 per decedent) (Fig 1). Only patients with chronic kidney disease incurred higher EOL costs than patients with IBD. Excess hospitalization cost was the major driver of excess EOL costs among patients with IBD and renal disease as compared to patients with four other chronic conditions associated with high morbidity and mortality–cancer, COPD, CHF and DM (S3 Table).

### Cost trajectory in the last year of life

**Fig 2:**
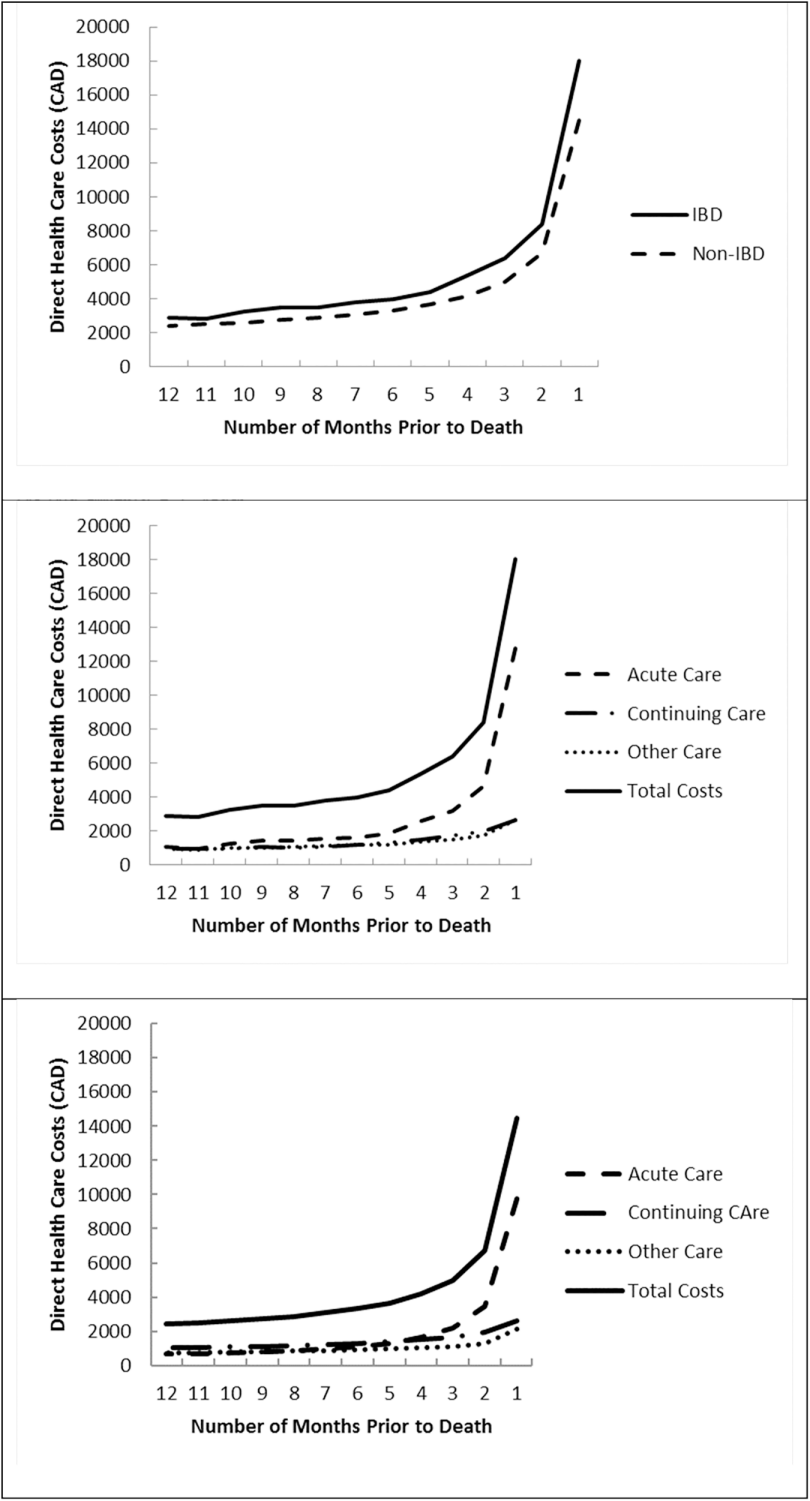
Mean individual direct health care costs per month over the last year of life. Top Panel: Total costs in IBD and non-IBD patients. Middle Panel: Total and sector-specific costs in IBD patients. Bottom Panel: Total and sector-specific costs in non-IBD patients.

Total direct health care costs per person rose steadily throughout the last year of life and sharply in the last 90 days (Fig 2). Costs for the last 90 days were 3.4 times higher than those for the first 90 days ($32,767 vs. $9,671 in IBD patients and $26,202 vs. $7,760 in non-IBD patients). Total costs over the last year and the rise in costs as patients approached death, were consistently higher in IBD as compared to non-IBD patients and were largely attributable to hospitalization costs.

### Specific health care use and costs in the last 90 days of life

IBD patients spent more time in institutional supportive care (acute care, complex continuing care, and rehabilitation) during the last 90 days of life than non-IBD patients (19.8vs. 15.2days), mainly due to longer acute hospital care (15.8 vs. 11.9 days). Crohn’s disease patients spent on average 1.5 more days in hospital than ulcerative colitis patients during the last 90 days of life (16.7 vs. 15.2 days).

After adjusting for socio-demographic factors and co-morbidity burden, IBD patients spent an additional 2.1 days (95% CI 1.4–2.8 days) in hospital during the last 90 days of life (Table 4). After excluding patients who died from external causes or complications of pregnancy or the puerperium, IBD diagnosis was associated with 2.3 more days spent in hospital (95% CI 1.6–3.0 days) during the last 90 days of life.

**Table 4:** Multiple linear regression model of number of hospital days per person in the last 90 days of life.

Variable	Parameter Estimate (95% CI)
IBD (vs. no IBD)	2.1 (1.4, 2.8)
Age Group	
<19	3.7 (2.8, 4.7)
19–44	0.15 (-0.27, 0.56)
45–54	-0.08 (-0.42, 0.26)
65–74	-0.32 (-0.57, -0.067)
75–84	-1.8 (-2.0, -1.6)
85–94	-4.5 (-4.7, -4.2)
95+	-6.5 (-6.8, -6.2)
(Ref 55–64)	
Female Sex (vs. Male)	0.17 (0.042, 0.30)
Income Quintile	
Q1	0.31 (0.11, 0.51)
Q2	0.34 (0.14, 0.54)
Q4	0.050 (-0.15, 0.26)
Q5	-0.30 (-0.51, -0.088)
(Ref Q3)	
Urban resident (vs. Rural)	0.77 (0.59, 0.95)
# ADG Score	0.41 (0.41, 0.42)

More than twice as many hospital admissions associated with MAS were observed among IBD patients in the last 90 days of life (0.064 vs. 0.029 per decedent), which was directly proportional to the difference in mean cost per person for such admissions during this period ($2,613 vs. 1,281 per decedent in IBD vs. non-IBD). However, the mean cost of a surgical hospitalization was slightly higher among non-IBD decedents ($44,675 vs $41,030). Additionally, close to twice as many endoscopic procedures (0.26 vs 0.14 per decedent) and 30% more advanced radiographic imaging studies were performed in IBD decedents (3.4 vs. 2.6 per decedent).

## Discussion

In this large population-based study of Ontario decedents, we have demonstrated that the EOL period represents a time of heightened health care resource use for IBD patients–markedly higher than for earlier periods of disease and considerably greater than for decedents in the general population. IBD patients incurred 23% higher health care costs per person during the last year of life and spent 2.1 more days in hospital during the last 90 days of life as compared to individuals without IBD in this study. Hospitalization cost was the major driver of EOL health care costs, accounting for 44% of total EOL costs and 76% of the cost differential between IBD and non-IBD decedents. Some of the major drivers of excess EOL hospitalization costs in IBD vs. non-IBD patients include longer length of hospital stay (LOHS), increased ICU burden and a greater frequency of major abdominal surgeries. However, a number of other factors, such as drug costs and costs of other medical illnesses, may also play a role.

EOL costs were higher in IBD patients in virtually all sectors of health care expenditures, and this closely matched health care resource usage in each sector. Astoundingly, the average cost of IBD patient care in the last year of life ($66,263 CAD) was even 13 times greater than the average annual cost of IBD patient care in Canada in 2012 ($5,192 CAD).[2] The excess cost of care in the last year of life for the 2,214 IBD patients in this study was more than $28 million CAD greater than EOL costs for the same number of patients in the general population. Health care costs in the last year of life were also 8% higher, on average, in IBD patients as compared to patients with other major chronic diseases, and this, again, was driven by excess hospitalization costs in IBD patients.

This is the first study to report on EOL health care costs and utilization in IBD patients and also one of the first studies to compare costs of care between IBD and non-IBD patients. We assessed a wide breadth of health care sectors and used a detailed case-costing methodology, while accounting for the effects of patient characteristics on health care use and costs and testing the robustness of the associations using several sensitivity analyses. As such, the study observations should present a relatively unbiased and comprehensive view of the impact of EOL IBD care on the health care system. This is particularly relevant given the already high contribution of EOL costs to the overall health care budget across the population (~10% of total direct health care costs), which is likely to increase in future years with aging baby boomers and increasing life expectancy.[10] While the Canadian health care system may differ from other countries, the EOL costs determined in this study should reasonably apply to other jurisdictions that have a largely public-payer health care system, such as developed countries in Western Europe, Australia and New Zealand. Importantly, the public payer only subsidises a portion of prescription drug costs in Ontario and other Canadian provinces–specifically those incurred by individuals 65 years of age and over, those requiring social assistance and those requiring support for very expensive drugs (such as biologic drugs, which are considered “exceptional access” drugs). Therefore, public payer costs may be higher in countries with national or regional pharma care programs. Conversely, our results may be less generalizable to jurisdictions with privatized or two-tier health care systems, such as the U.S. and many countries in Asia as well as developing countries. Nevertheless, our findings may reasonably reflect the overall health care costs borne by third party payers even in these jurisdictions.

Other studies in IBD cohorts outside of the EOL period have reported that hospitalizations and medical therapy account for 30–45% and 30–40% of direct health care costs, respectively.[2;4–6] In our study, prescription drug costs constituted only 5.5% of IBD-related health care costs. This may partly relate to restricted ascertainment of drug costs to patients aged 65 and over, those receiving government assistance and those requiring assistance with payment for very expensive medications. However, more than 72% of IBD patients were 65 or older and many younger patients without private insurance would have received government assistance for expensive biologic drugs; therefore, the majority of IBD-related drug costs should have been captured in the OHIP databse. Notably, biologic agents, which are typically the main driver of medication costs in IBD patients, are used less frequently in elderly IBD patients, and this may have led to a lower contribution of drug costs to overall health care costs at the EOL.[25] Biologic drugs accounted for less than 11% of all prescription drug costs in our cohort. It is equally possible that the rise in drug costs is simply outpaced by the rise in institutional costs as IBD patients approach the EOL. As less than 12% of deaths among IBD patients were reported to be related to IBD itself (accepting some inevitable misclassification in health administrative data), other medical illnesses may have largely driven hospitalization costs at the EOL in IBD patients. Importantly, even after excluding the costs of drugs and devices, IBD diagnosis was associated with greater than $6,000 higher individual health care costs in the last year of life.

The factors driving greater EOL health care use and costs across various sectors in IBD patients are likely complex and comprise both IBD and non-IBD indications. As only a minority of IBD patients actually had IBD listed as a most responsible or co-morbid cause of death in this study, IBD burden could have contributed to a higher cost of dying from other illnesses. IBD may be considered a more treatable illness as compared to other chronic diseases, which may lead practitioners to offer expensive acute care services and medical and surgical therapies directed at active IBD, even in the face of deteriorating illness from other causes. On the other hand, when faced with progressive deterioration of IBD itself, practitioners may find it difficult to gauge when death is inevitable and further complex treatments are likely to be futile, leading to escalating use of acute services with progressive illness. Inadequate access to outpatient specialist care as well as palliative care services may also contribute to greater use of hospital resources at the EOL in these patients. Further studies are required to better characterize preventable causes of higher health care sector costs in these patients at the EOL.

There are several limitations to our study. First, cost estimates in this study captured only about 80% of total direct public payer health care costs and did not capture out-of-pocked expenses or indirect costs associated with lost productivity.[2] Second, costs of prescription drugs and devices were not comprehensively captured for 20% of patients (those under the age of 65). Third, misclassification of some patients may have impacted the association between IBD diagnosis and study outcomes. We anticipate that the extent of misclassification would be small as we used a validated algorithm to ascertain IBD patients from among all decedents. Fourth, we may not have captured all confounding influences on the studied relationships. Fifth, we were unable to fully assess specific factors contributing to higher health care sector costs and acute care use among IBD patients in this study. We acknowledge that this is an important goal of future work in this area. Sixth, although we believe that our results give an accurate representation of the increase in both health care demands and associated costs for patients with IBD in the last year of life, our cost estimates may not be fully generalizable to all health care funding models.

In summary, we have shown that EOL direct health care costs and acute care use in IBD patients are significantly higher than in patients without IBD, including those with other major chronic diseases, studying a wide breadth of health care sectors and using detailed case-costing methods. We found that IBD decedents use virtually all aspects of the health care system more than non-IBD decedents, but that excess hospitalization burden is the major cost driver at the EOL. However, the extent to which excess EOL costs are preventable in IBD patients, such as through avoidance of unnecessary testing and treatment and shifting of care from an inpatient to an outpatient setting with palliative supports, in the face of irreversible deterioration of health, is unclear. Future studies should evaluate modifiable health care sector costs, particularly hospitalization costs, at the EOL in IBD patients, as well as factors that predict entry into the EOL period in this patient population.

## Supporting information

S1 Table Databases used to record health care use and cost at the end-of-life. *CIHI-DAD: Canadian Institute for Health Information-Discharge Abstract Database.
(DOCX)

S2 Table Physician fee codes used to identify relevant major abdominal surgeries, endoscopies and abdominal imaging studies from the OHIP database for comparison between IBD and non- IBD patients in the last 90 days of life*. *Relevant codes were identified from the 2013 Ontario Schedule of Benefits for Physician Services (available in the Public Domain on-line); aIncludes intra-abdominal surgeries on stomach, intestines, appendix and peritoneum that are potentially relevant to IBD; bIncludes all gastroscopies, colonoscopies, endoscopic retrograde cholangiopancreatography and endoscopic ultrasound procedures; cIncludes computed tomography and magnetic resonance imaging studies of the head, chest, abdomen and/or pelvis, ultrasound examinations of the chest and/or abdomen and complete echocardiography.
(DOCX)

S3 Table Mean health care sector costs per decedent during the last year of life across individuals with common chronic diseases. CHF = Congestive Heart Failure; COPD = Chronic Obstructive Pulmonary Disease; CRF = Chronic Renal Failure.
(DOCX)
