# Supplementary material for: High end of life health care costs and hospitalization burden in inflammatory bowel disease patients: A population-based study
Source: PLoS One. 2017 May 12;12(5):e0177211. doi: 10.1371/journal.pone.0177211 (PMC5428925; doi:10.1371/journal.pone.0177211)
Supplement: S1 Table — *CIHI-DAD: Canadian Institute for Health Information-Discharge Abstract Database. (DOCX) [file pone.0177211.s001.docx]

| **Supplemental** **Table 1:** Databases used to record health care use and cost at the end-of-life | | |
| --- | --- | --- |
| Health care Sector | Database | Description |
| **Continuing Care** | | |
| Long-term Care | Continuing Care Reporting System (CCRS) | Population-based resident information for over 600 publicly funded residential care homes with 24-hour nursing care |
| Complex Continuing Care | CCRS | Population-based information for all patients staying in a designated complex continuing care bed. These individuals are typically deemed to be in a non-acute state, but still in need for treatment (e.g., rehabilitation) in an institution |
| Home Care | Home Care Database (HCD)  Resident Assessment Instrument-Home Care (RAI-HC) | Data from the Ontario Association of Community Care Access Centers, responsible for providing all publicly funded home care |
| Rehabilitation | National Rehabilitation Reporting System (NRS) | Data from participating adult inpatient rehabilitation facilities and programs across Ontario |
| **Acute Care** | | |
| Inpatient without ICU | CIHI-DAD* | Administrative, clinical, and demographic data on all hospital discharges in Ontario |
| Inpatient with ICU | CIHI-DAD | Individuals with at least one Intensive Care Unit (ICU) visit in their last year of life |
| Emergency Department | National Ambulatory Care Reporting System (NACRS) | Captures all emergency department visits in Ontario |
| **Outpatient Care** | | |
| Outpatient clinics | NACRS | Select outpatient visits held in hospitals, including dialysis clinics and cancer care clinics |
| Physician Billings | Ontario Health Insurance Plan (OHIP) Claims Database | Claims data for physicians in Ontario – includes claims in both inpatient and outpatient settings. |
| Non-physician Billings | OHIP | Health professionals for provincially insured services, such as select midwives, oral surgeons, chiropractors, optometrists, and physiotherapists. Some care may occur for inpatients |
| Laboratory | OHIP | Outpatient laboratory services. Does not include laboratory services for inpatients |
| Drugs/Devices | Ontario Drug Benefit (ODB), Assistive Devices Program (ADP) | Drugs for those over 65 years, on social assistance, residents of LTC, home care recipients, Trillium drug program and special drugs program recipients for those qualifying for assistance. Select medically-necessary devices including home oxygen and respiratory devices. |
| *CIHI-DAD: Canadian Institute for Health Information-Discharge Abstract Database  Adapted from Tanuseputro et al.^22^ with permission from P. Tanuseputro | | |
