# Supplementary material for: High end of life health care costs and hospitalization burden in inflammatory bowel disease patients: A population-based study
Source: PLoS One. 2017 May 12;12(5):e0177211. doi: 10.1371/journal.pone.0177211 (PMC5428925; doi:10.1371/journal.pone.0177211)
Supplement: S2 Table — *Relevant codes were identified from the 2013 Ontario Schedule of Benefits for Physician Services (available in the Public Domain on-line); aIncludes intra-abdominal surgeries on stomach, intestines, appendix and peritoneum that are potentially relevant to IBD; bIncludes all gastroscopies, colonoscopies, endoscopic retrograde cholangiopancreatography and endoscopic ultrasound procedures; cIncludes computed tomography and magnetic resonance imaging studies of the head, chest, abdomen and/or pelvis, ultrasound examinations of the chest and/or abdomen and complete echocardiography. (DOCX) [file pone.0177211.s002.docx]

| **Supplemental Table 2**: Physician Fee Codes Used to Identify Relevant Major Abdominal Surgeries, Endoscopies and Abdominal Imaging Studies from the OHIP Database for Comparison between IBD and non- IBD Patients in the Last 90 Days of Life* | |
| --- | --- |
| Category | OHIP Codes |
| Major Abdominal Surgeries^a^ | S122 S123 S125 S128 S129 S132 S133 S137 S134 S138 S139 S140 S149 S150 S151 S154 S155 S156 S157 S158 S160 S162 Z750 S164 S165 S166 S167 S169 S172 S171 S168 S170 S173 S174 S188 S175 S176 S177 S180 S181 S182 S192 S191 S193 S183 S184 S185 S187 S194 S159 S195 S199 S204 S205 S206 S213 S214 S215 S216 S217 S218 S222 S223 S229 S231 S525 S251 S313 S321 S314 Z569 Z594 S312 Z564 S311 |
| Endoscopic Studies^b^ | Z515 Z399 Z400 Z527 Z547 Z528 Z560 Z749 Z584 Z512 Z514 Z580 Z497 Z499 Z492 Z493 Z496 Z494 Z498 Z495 Z491 Z555 Z561 Z558 Z760 S236 S237 |
| Abdominal Imaging Studies^c^ | X400 X401 X188 X402 X405 X408 X404 X124 X406 X407 X125 X235 X409 X410 X126 X231 X232 X233 X234 X412 X413 X127 X415 X416 X128 X168 X417 X421 X425 X431 X435 X441 X445 X451 X455 X480 X481 X446 X447 X461 X499 X471 X475 X488 X489 X490 X492 X493 X495 X496 X498 X465 J125 J425 J135 J435 J128 J428 G570 G572 G571 G582 G585 G570 G577 G585 |

*Relevant codes were identified from the 2013 Ontario Schedule of Benefits for Physician Services (available in the Public Domain on-line)

^a^Includes intra-abdominal surgeries on stomach, intestines, appendix and peritoneum that are potentially relevant to IBD

^b^Includes all gastroscopies, colonoscopies, endoscopic retrograde cholangiopancreatography and endoscopic ultrasound procedures

^c^Includes computed tomography and magnetic resonance imaging studies of the head, chest, abdomen and/or pelvis, ultrasound examinations of the chest and/or abdomen and complete echocardiography
