# Supplementary material for: High end of life health care costs and hospitalization burden in inflammatory bowel disease patients: A population-based study
Source: PLoS One. 2017 May 12;12(5):e0177211. doi: 10.1371/journal.pone.0177211 (PMC5428925; doi:10.1371/journal.pone.0177211)
Supplement: S3 Table — CHF = Congestive Heart Failure; COPD = Chronic Obstructive Pulmonary Disease; CRF = Chronic Renal Failure. (DOCX) [file pone.0177211.s003.docx]

| **Supplemental Table 3: Mean Health Care Sector Costs Per Decedent During the Last Year of Life Across Individuals with Common Chronic Diseases** | | | | | |
| --- | --- | --- | --- | --- | --- |
|  | **Mean Cost per decedent ($CAD)** | | | | |
| **Health Care Sector** | **Diabetes (N=93,064)** | **CHF (N=88,641)** | **COPD (N=65,984)** | **CRF (N=62,343)** | **Cancer (N=104,600)** |
| All-Cause Hospitalization | 28435.78 | 29779.34 | 28416.79 | 35588.83 | 26702.17 |
| Emergency Department | 1465.6 | 1593.36 | 1629.79 | 1719.68 | 1493.88 |
| Complex Continuing Care | 4090.97 | 4146.49 | 3882.1 | 4420.38 | 3547.18 |
| Rehabilitation | 1111.51 | 1262.11 | 1064.47 | 1448.07 | 1035.46 |
| Long-term Care | 8362.31 | 8979.74 | 7948.64 | 7393.5 | 2674.68 |
| Home Care | 4878.82 | 5013.8 | 4952.52 | 5266.17 | 5907.21 |
| Outpatient clinics | 4189.23 | 3325.57 | 3036.31 | 6149.11 | 6553 |
| Physician Billings | 6128.06 | 6038.67 | 5855.2 | 7199.77 | 7078.1 |
| Non-physician Billings (OHIP) | 321.36 | 367.72 | 310.04 | 314.13 | 148.12 |
| Laboratory (OHIP) | 269.83 | 286.56 | 238.77 | 301.18 | 212.69 |
| Drugs/Devices | 3531.05 | 3359.97 | 3642.89 | 3459.27 | 3484.48 |
| **Total Cost** | 62784.52 | 64153.32 | 60977.51 | 73260.1 | 58836.96 |

CHF = Congestive Heart Failure; COPD = Chronic Obstructive Pulmonary Disease; CRF = Chronic Renal Failure
